# Machine learning-assisted tongue image analysis for the diagnosis of Hashimoto’s thyroiditis

**DOI:** 10.3389/fmed.2025.1673891

**Published:** 2025-10-29

**Authors:** Ting Ruan, Wenjun Wu, Mingji Piao, Yihan Sun, Xingai Ju, Mengyou Liu, Li Lu, Bo Zhang, Yifei Zeng, Dongxiao Zhang, Yongxin Li, Jianchun Cui

**Affiliations:** ^1^Liaoning University of Traditional Chinese Medicine, Shenyang, China; ^2^Changchun University of Traditional Chinese Medicine, Changchun, China; ^3^Department of General Surgery, Affiliated Hospital of Liaoning University of Traditional Chinese Medicine, Shenyang, China; ^4^Department of Cardiology, People's Hospital of China Medical University (People's Hospital of Liaoning Province), Shenyang, China; ^5^Department of General Medicine, People's Hospital of China Medical University (People's Hospital of Liaoning Province), Shenyang, China; ^6^Department of Thyroid and Breast Surgery, Lixin County People's Hospital, Lixin, Anhui, China; ^7^Department of Endocrinology, People's Hospital of China Medical University (People's Hospital of Liaoning Province), Shenyang, China; ^8^The 10th Division Beitun Hospital of Xinjiang Production and Construction Corps, Beitun, Xinjiang, China; ^9^Beijing Hospital of Traditional Chinese Medicine, Capital Medical University, Beijing, China; ^10^Department of Research and Development, Yizhun Medical AI Co, Ltd., Beijing, China; ^11^Department of Thyroid and Breast Surgery, People's Hospital of China Medical University (People's Hospital of Liaoning Province), Shenyang, China

**Keywords:** tongue image, machine learning, Hashimoto’s thyroiditis, hypothyroidism, AI-assisted diagnosis

## Abstract

**Objective:**

This study aims to evaluate the value of a machine learning model based on tongue features in the adjunctive diagnosis of Hashimoto’s thyroiditis (HT) and its concomitant hypothyroidism.

**Methods:**

Tongue images and related clinical data were retrospectively collected from 120 HT patients (60 each from the euthyroid group and the hypothyroidism group), and the tongue region was segmented by preprocessing, and the image feature dimensions were extracted with 1,125 dimensions. Therefore, four methods, namely, random forest (RF), logistic regression (LR), support vector machine (SVM), and decision tree (DT), were utilized for model training, and 80 tongue images of 40 patients from Lixin County People’s Hospital in Anhui Province were utilized for external validation. The model performance evaluation indexes included AUC (Area Under the Curve), Sensitivity, Specificity, Positive Predictive Value (PPV), and Negative Predictive Value (NPV).

**Results:**

t-Distributed Stochastic Neighbor Embedding (t-SNE) visualization based on the test set revealed a distinguishable clustering trend between the two groups. The key classification features included tongue texture uniformity, body morphological features, and color depth. The AUC of the four models was higher than 0.82, confirming that the tongue image features have significant predictive value for HT, and the lower limit of 95% CI for all models was higher than 0.75, indicating that the models had stable differentiation ability. The AUC of SVM (0.894) was the best, significantly higher than the other models (RF: 0.857, LR: 0.876, and DT:0.828), indicating that the SVM possesses the strongest ability to classify patients with and without HT and the highest stability. The SVM exhibited balanced performance, with a sensitivity of 0.804 and specificity of 0.936. Consequently, it represents the optimal model for achieving an equilibrium between recall and precision. In external validation, the efficacy of the four models is notable, and the trend is consistent with the test set. SVM still demonstrates notable performance and possesses the best generalization ability among the four models.

**Conclusion:**

The tongue image-based machine learning model can effectively assist in distinguishing euthyroid from hypothyroidism in HT, offering a non-invasive, low-cost, and intelligent tool for auxiliary diagnosis and disease risk monitoring in primary care settings.

## 1 Introduction

Primary hypothyroidism affects more than 40 million patients in China, of which 80% are cautilized by Hashimoto’s thyroiditis (HT) (1). Therefore, HT and its associated hypothyroid states usually present with an insidious onset of disease and nonspecific symptoms, leading to substantial underdiagnosis that adversely affects patient quality of life and long-term health outcomes (2). Currently, the diagnosis of HT relies on thyroglobulin (TgAb) and thyroid peroxidase antibody (TPOAb) detection, yet approximately 20% of HT patients are antibody-negative, resulting in a significant underdiagnosis risk (3). Although fine-needle aspiration biopsy (FNAB) of the thyroid gland may be utilized as a basis for confirming the diagnosis, which is mainly characterized by lymphocytic infiltration of thyroid tissue and cytoplasmic eosinophilic changes in follicular epithelial cells, its invasive nature limits clinical adoption (4)^.^ Ultrasonography serves as a noninvasive adjunct but has limited accuracy in differentiating euthyroid from hypothyroid states (5).

Tongue diagnosis represents a pivotal non-invasive diagnostic technique in both traditional Chinese medicine (TCM) and Western medical practice. Rooted in TCM theory that “all illnesses within the body must be manifested outside,” tongue alterations reflect systemic physiological and pathological states with significant specificity and quantifiability. Variations in the tongue’s color, morphology, coating, and texture sensitively indicate disease etiology, nature, and progression stage. In recent years, tongue features have been gradually digitized as extractable image features and applied to the diagnosis and monitoring of various diseases (6–8). Tongue image analysis has been demonstrated to assist in assessing the risk of malignancy in thyroid nodules (9), and its morphological markers have demonstrated an important role in screening for various chronic diseases such as diabetes mellitus, non-alcoholic fatty liver disease, and COVID-19 (10–12) Therefore, identifying characteristic tongue features in HT and associated hypothyroidism, and constructing a tongue classification model by machine learning, possesses important clinical research and translational application value.

In Recent years, we have witnessed numerous machine learning applications for precise clinical image analysis, enabling the construction of imaging-based screening, diagnostic, and risk prediction models (13, 14). At present, the application of machine learning in TCM tongue diagnosis mainly focuses on standardizing tongue image processing, building image databases, and developing prediction models, which helps to reduce the bias brought by subjective judgment in the traditional manual tongue diagnosis, and improve the objectivity and consistency of diagnosis (15, 16). However, Limited research exists on machine learning-assisted tongue diagnosis for Hashimoto’s thyroiditis (HT) and concomitant hypothyroidism. Based on this study aimed to develop an auxiliary diagnostic model integrating tongue image features and machine learning to effectively differentiate between euthyroid and hypothyroid states among patients already diagnosed with HT. This distinction is crucial for disease management, particularly in deciding whether to initiate or adjust levothyroxine therapy. Thus, this work provides a non-invasive, low-cost, and easily deployable intelligent auxiliary screening tool for primary healthcare settings.

## 2 Materials and methods

### 2.1 Patient enrollment and data classification

This retrospective study received ethics approval from Liaoning Provincial People’s Hospital (no. 2023H014) and Lixin County People’s Hospital, Anhui Province (no. LXXRMYY-2025KY015), with waiver of informed consent. We collected clinical data and tongue images from 120 HT patients at People’s Hospital of Liaoning Province (January 2023–March 2024) as the training set. The ratio of the training set to the test set was 8:2. Owing to the retrospective nature of this study, no formal *a priori* power calculation was conducted. However, a *post hoc* power analysis was conducted using a target effect size (AUC of 0.85, indicating high discrimination), a significance level (*α*) of 0.05, a statistical power of 0.8, and a 1:1 sample allocation ratio; this estimation indicated that a minimum of 30–50 samples per group would be required. Consequently, our training cohort, which comprised 60 patients per group, exceeded this minimum sample size requirement. Furthermore, an independent external validation set was established, consisting of 40 patients recruited from Lixin County People’s Hospital in Anhui Province. Rigorous privacy-protection protocols were implemented for all patient data, including clinical records and tongue images, to ensure anonymity and prevent re-identification. All potentially identifiable information (e.g., hands, apparel) was cropped or obscured from the images, which were then irreversibly de-identified prior to subsequent processing and storage. All data were maintained on a secure, password-encrypted server with access restricted exclusively to authorized research staff. All tongue images underwent quality control, excluding blurred or incomplete specimens. Patients were stratified by thyroid function tests: euthyroid or hypothyroid groups. Electronic records provided additional covariates: demographics (sex, age) and levothyroxine treatment (duration, dosage). These parameters formed two labeled data categories.

*Inclusion criteria*: HT patients who met the diagnostic criteria of the 2008 Chinese Guidelines for the Diagnosis and Treatment of Thyroid Diseases - Thyroiditis (17). Stringent exclusion criteria were applied to control for confounding factors from medications and comorbidities: pregnancy; thyroid surgery history, post I131 therapy for hyperthyroidism, Graves’ disease (TRAb-positive), and subacute thyroiditis; current/past thyroid-affecting medications (e.g., glucocorticosteroids, amiodarone, lithium, oral contraceptives, metformin, etc.); obesity [BMI (Body Mass Index) > 28 kg/m^2^]; combination of severe cardiac, brain, liver, kidney and hematopoietic system underlying diseases.

**Figure 1:**
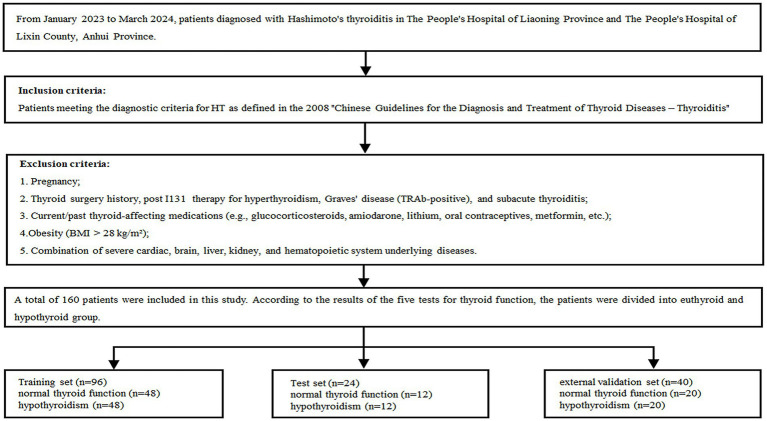
The cohort flow diagram.

Four standardized tongue images (frontal, left lateral, right lateral, and basal views) were acquired per patient. All images were captured under controlled lighting and positioning using identical equipment/parameters to ensure consistency. The cohort flow diagram appears in Figure 1.

### 2.2 Data pre-processing

All tongue images were in PNG format. Images were imported into the Beijing Yizhun AI Darwin Research Platform, and the region of interest (ROI) was manually delineated by an experienced TCM practitioner and independently reviewed by a senior practitioner (20 years of experience). The image standardization was ensured by size normalization and color correction; the tongue features were normalized by maximum absolute value normalization; and the minimum redundancy maximum relevance (mRMR) (18) method was employed for feature selection, an advanced filter-based feature selection technique that goes beyond conventional dimensionality reduction. This method aims to identify a subset of features from the initial 1,125-dimensional space that maximizes relevance to HT status while minimizing inter-feature redundancy. By balancing discriminative power and redundancy, the mRMR algorithm effectively reduces the risk of overfitting inherent in high-dimensional datasets, thereby improving model performance and generalizability. The preprocessed tongue image dataset is formed from the above steps.

### 2.3 Model construction and evaluation

**Figure 2:**
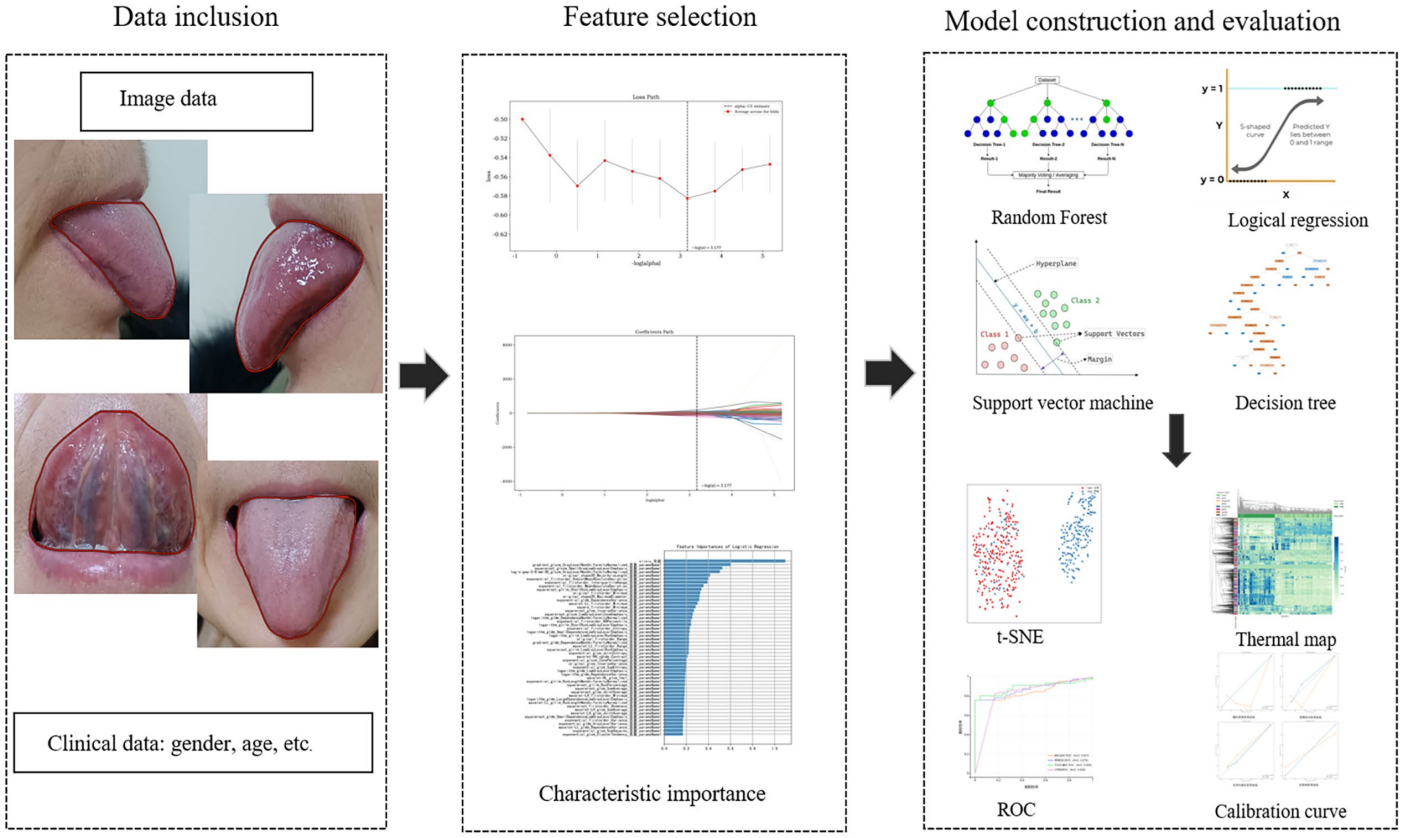
The study framework diagram.

Based on the characteristics of the data, machine learning algorithms were employed to develop a radiomics model. Specifically, for model selection, we chose four models: Random Forest (RF), Logistic Regression (LR), Support Vector Machine (SVM), and Decision Tree (DT). These models were selected because they are commonly used as baseline models in current research and possess representativeness. RF integrates multiple independently trained decision trees, outputting predictions through majority voting to confer strong overfitting resistance and nonlinear modeling capacity. LR applies logistic functions to model relationships between dependent and independent variables, outputting event probabilities for binary classification. SVM constructs optimal hyperplanes to maximize inter-category margins to achieve the optimal division of data points, which is especially suitable for classification tasks with small samples and high-dimensional problems (19). DT constructs a classification structure based on a recursive splitting strategy, which can be demonstrated as a probabilistic tree based on the statistical significance separation results, and the optimal feature nodes can be selected by criteria such as information gain or Gini index, which possesses notable interpretability (20). The area under the curve (AUC) is utilized to measure the overall discriminative ability of the model. Meanwhile, other evaluation metrics were derived from confusion matrices, including sensitivity, specificity, positive predictive value (PPV), and negative predictive value (NPV). The research framework diagram is detailed in Figure 2.

### 2.4 Statistical analysis

The statistical analysis was performed using SPSS software (version 26.0). Before analysis, all data were assessed for normality and homogeneity of variance. Normally distributed quantitative data are presented as the mean ± standard deviation (x ± s), while non-normally distributed data are expressed as the median. For quantitative variables, the *t*-test or Mann–Whitney U test was applied, depending on the distribution. For categorical data, the Pearson *χ*^2^ test or Fisher’s exact test was used. Quantitative results are reported as the mean ± standard deviation (SD), and a *p* < 0.05 was considered statistically significant for all tests.

## 3 Results

### 3.1 General clinical data

This retrospective multicenter study included 160 patients’ clinical data and 555 tongue images from People’s Hospital of Liaoning Province (training set) and Lixin County People’s Hospital, Anhui Province (external validation set). As a retrospective cross-sectional study, no strict matching for age, sex, or disease duration was performed. However, the baseline characteristics presented in Table 1 indicate that although there was a predominance of female patients in the HT cohort, nevertheless, no statistically significant differences were observed in age or sex distribution between the euthyroid and hypothyroid groups within either the internal training set or the external validation set. Consistent with hypothyroidism pathophysiology, significant reductions in FT3 (Free Triiodothyronine) and FT4 (Free Thyroxine) with elevated TSH (Thyroid-Stimulating Hormone) were observed across both cohorts (*p* < 0.05), indicating that the study subjects are well comparable on other clinical backgrounds, offering a basis for subsequent modeling of image features.

**Table 1:** Baseline characteristics of HT patients: euthyroid vs. hypothyroid.

Parameter	Internal training and verification set		*p*-value	External verification set		*P*-value
	Normal (*n* = 60)	Hypothyroid (*n* = 60)		Normal (*n* = 20)	Hypothyroid (*n* = 20)	
Age	55.0 (19.0–74.0)	56.0 (24.0–79.0)	0.086	47.5 (21.0–74.0)	47.5 (25.0–70.0)	0.787
Gender, *n* (%), (male/female)	5 (8%)/55 (92%)	4 (7%)/56 (93%)	0.729	0 (0%)/20 (100%)	1 (5%)/19 (95%)	0.311
FT3 (nmol/L)	3.2 (1.3–8.8)	3.0 (0.0–5.1)	<0.01 **	3.3 (2.7–5.6)	2.9 (1.7–3.8)	<0.01 **
FT4 (nmol/L)	1.2 (0.8–1000.0)	1.1 (0.1–9.2)	<0.01 **	1.2 (1.0–16.4)	1.1 (0.4–1.6)	<0.05*
TSH (mIU/L)	2.3 (0.0–5.5)	6.5 (0.0–137.3)	<0.01 **	1.6 (0.8–7.0)	5.2 (0.2–90.1)	<0.01 **
aTG (IU/mL)	17.8 (0.2–1000.0)	34.1 (0.2–1000.0)	0.461	2.2 (1.3–1000.0)	5.6 (0.2–458.4)	0.139
aTPO (IU/mL)	936.1 (0.7–1300.0)	1010.7 (28.0–2000.0)	0.864	93.5 (4.0–1300.0)	1300.0 (28.0–1300.0)	<0.05*

Qualitative variables are expressed in n (%), and continuous variables are expressed as median (range).The symbols * and ** are markers used to denote the significance level of p-values in statistical tests, with * indicating *p* < 0.05 and ** indicating *p* < 0.01. FT3 (free triiodothyronine); FT4 (free thyroxine); TSH (thyroid-stimulating hormone); aTPO (antithyroid peroxidase antibody); aTG (antithyroglobulin antibody).

### 3.2 Tongue feature recognition and visualization

**Figure 3:**
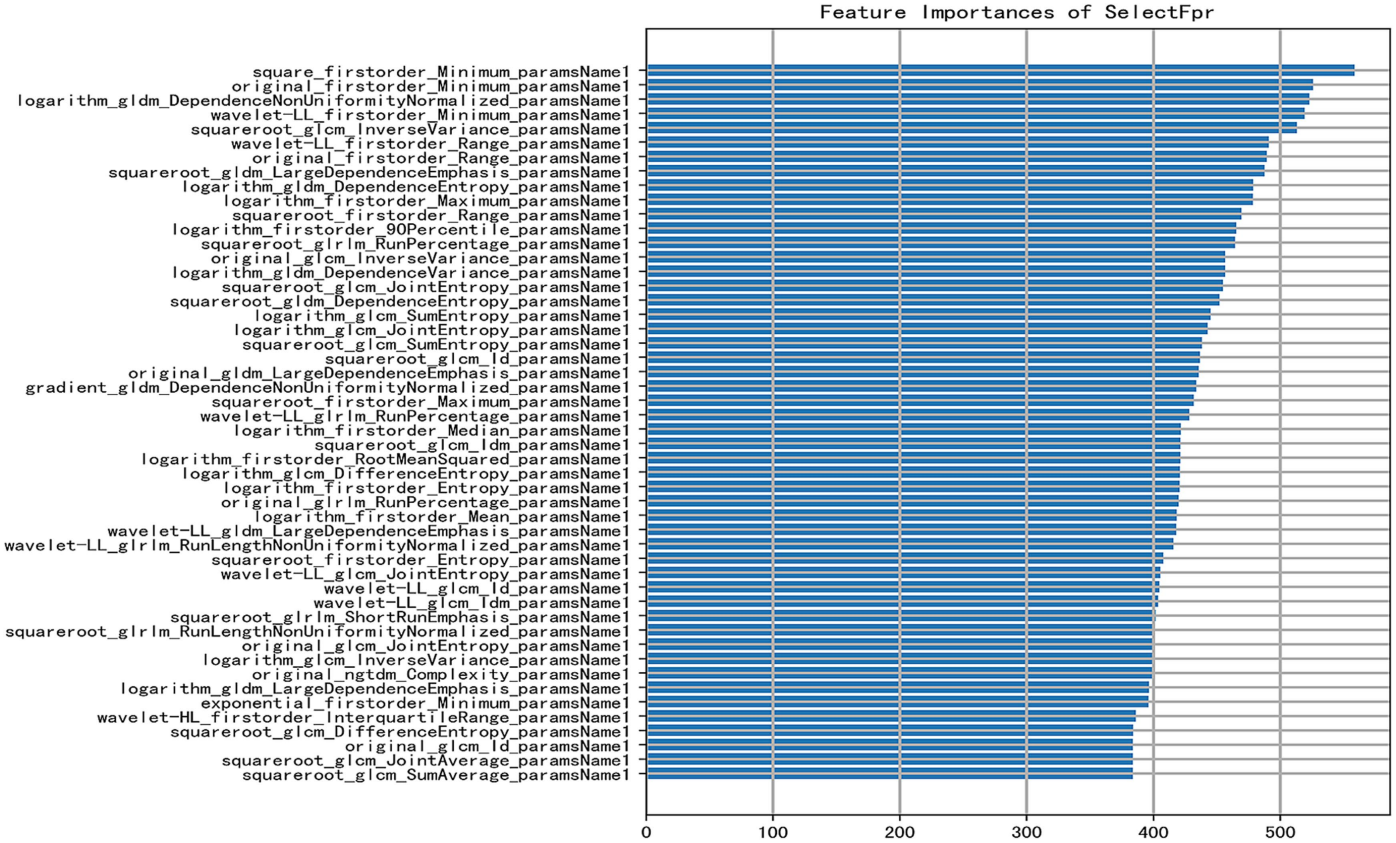
Bar plot of feature importance [the X-axis indicates the feature importance score (approximately 0–500); a higher score denotes a greater contribution of the feature to predicting HT. The Y-axis lists selected key features used in the model, ranked in descending order of importance. Each feature is represented by a horizontal bar reflecting its importance score].

Four machine learning methods, including random forest (RF), logistic regression (LR), support vector machine (SVM), and decision tree (DT), were utilized to process 1,125-dimensional clinical features from 475 samples and construct the model. The minimum Redundancy Maximum Relevance (mRMR) method was employed for feature selection. This process identified a subset of tongue image features encompassing texture, margin, chromaticity, and morphology, which were categorized as follows: First-order statistics capture the distribution of pixel intensities, indicating the global intensity characteristics of the tongue body. The Gray Level Co-occurrence Matrix (GLCM) characterizes the spatial relationships between pixel pairs, reflecting the roughness and textural homogeneity of the tongue coating. The Gray Level Size Zone Matrix (GLSZM) quantifies the size distribution of homogeneous regions, revealing the aggregation and dispersion patterns of the tongue coating. The Gray Level Dependence Matrix (GLDM) captures the local dependencies among pixels, corresponding to textural fineness. Two-dimensional shape descriptors define the geometric properties and contour of the tongue body (Shape 2D). These feature types collectively provide a comprehensive quantitative representation of tongue images, facilitating their processing by machine learning algorithms; notably, their importance differed significantly during modeling (Figure 3). First-order statistical features (e.g., Minimum, Range, Maximum, Entropy) appeared frequently, suggesting that the distribution of dark areas in tongue images holds significant diagnostic value for HT. Texture features (GLCM, GLDM, GLRLM) also contributed substantially, indicating that structural information—such as texture, homogeneity, and run-length of the tongue coating—possesses discriminatory power. The application of diverse preprocessing methods demonstrated that different transformations could extract useful information from various aspects of the images.

**Figure 4:**
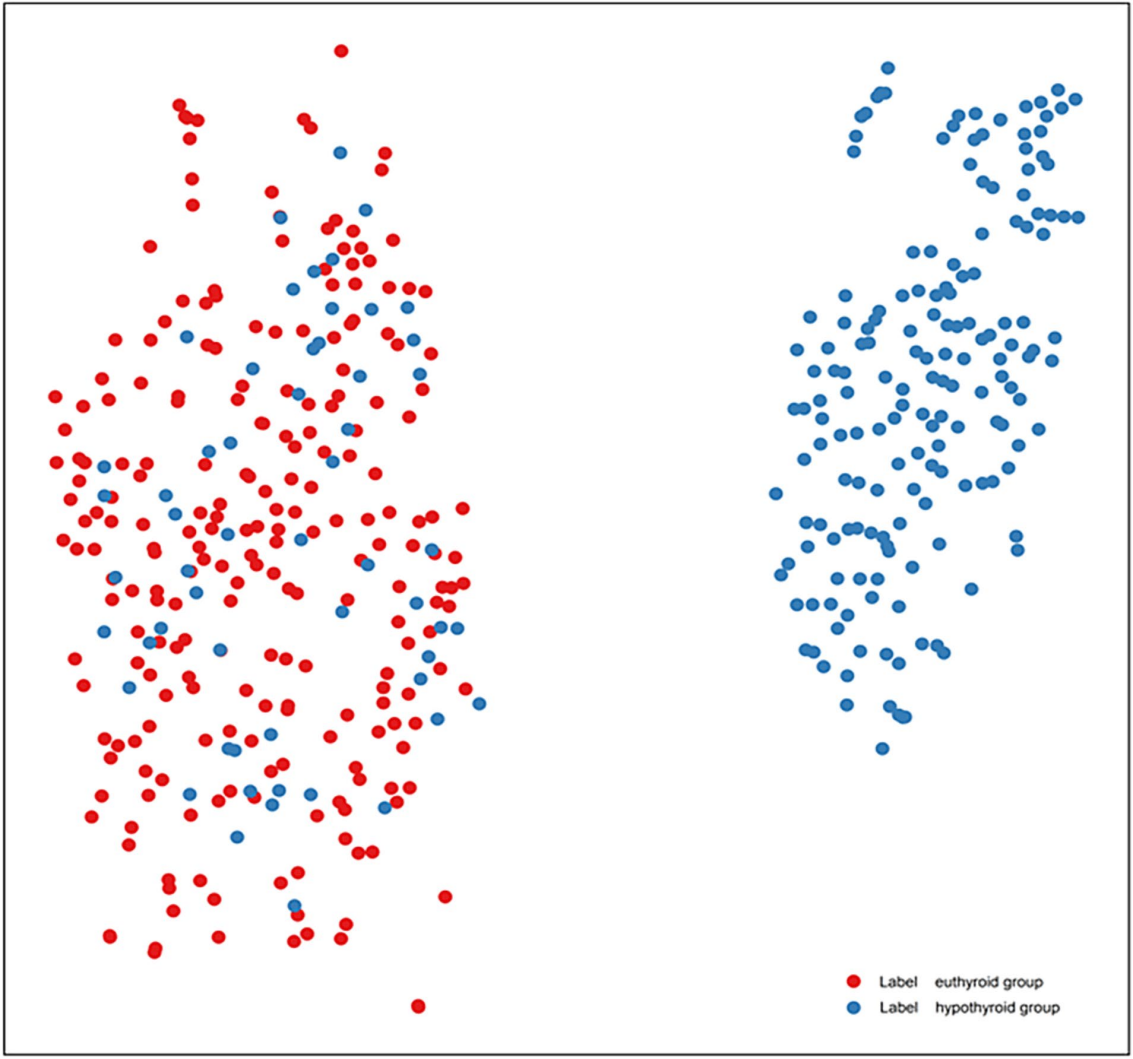
t-SNE visualization of tongue feature distributions in HT patients (lable red is euthyroid group, lable blue is hypothyroid group).

In addition to mRMR feature selection, the t-SNE algorithm was employed; however, it was used solely for visualization purposes to project the high-dimensional feature space onto a two-dimensional plane for intuitive visualization of data distribution and was not utilized for model training (Figure 4). Discernible separation trend was observed between euthyroid (red) and hypothyroid (blue) groups, confirming the significant discriminative capacity of tongue features and validating the classification model’s feasibility.

**Figure 5:**
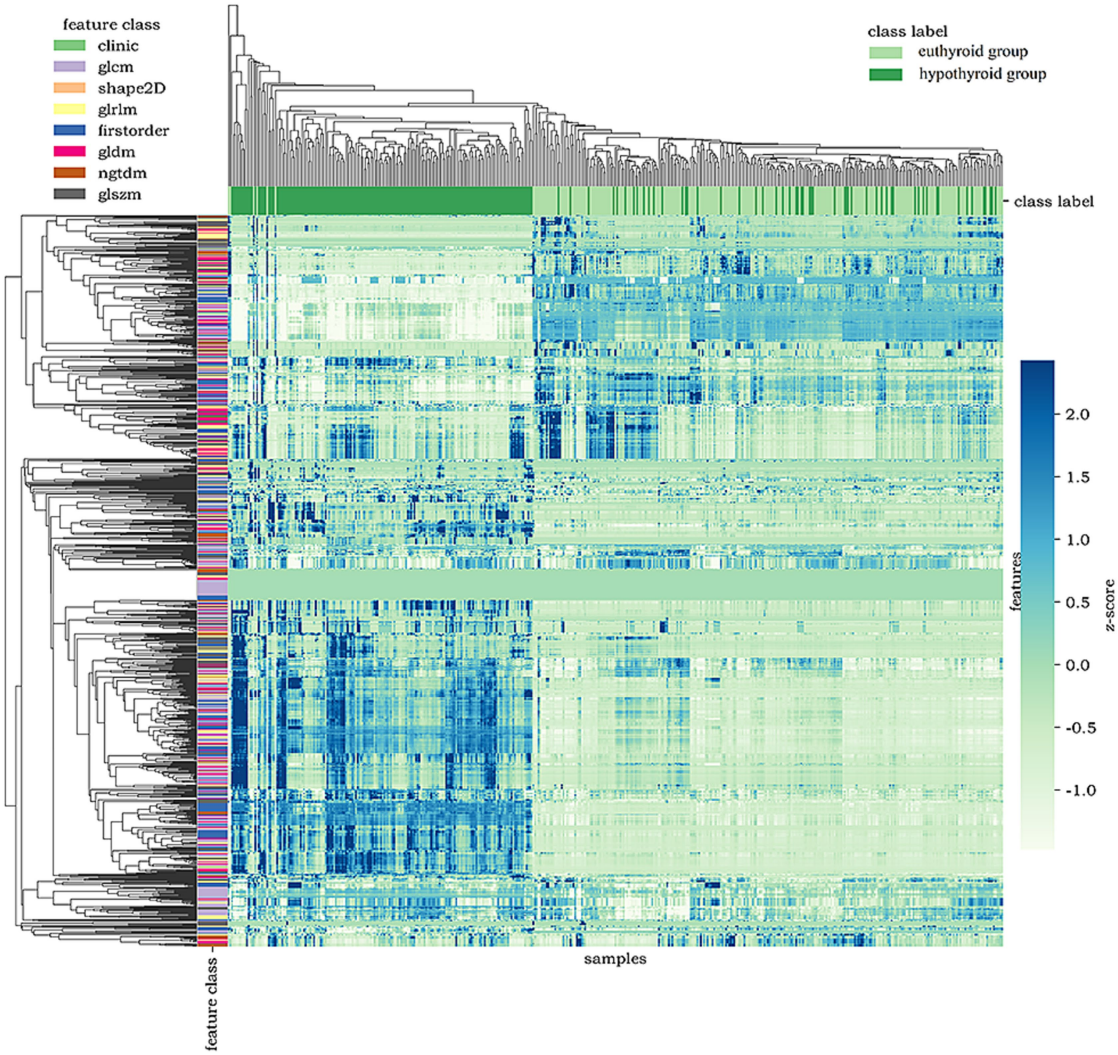
Heatmap of tongue image features.

Figure 5 presents a feature heatmap with samples on the horizontal axis, feature types on the vertical axis, and left-side color bars indicating feature categories. Samples are stratified by thyroid status (green and dark green bars above). Notably, directional features (GLCM, GLSZM, Shape2D) exhibit significantly distinct high-expression patterns.

### 3.3 Model diagnostic performance evaluation

Model performance in distinguishing euthyroid versus hypothyroid HT states was evaluated using AUC, sensitivity, specificity, PPV, NPV, and accuracy. Table 2 summarizes diagnostic performance across test and external validation sets. Test set analysis revealed that DT achieved the highest sensitivity (0.826), followed by SVM (0.804), with RF and LR showing lower sensitivity (0.761), and the upper limit of the 95% CI of DT amounted to 0.909, which indicated that it was best at identifying true-positive cases. Specificity, which indicates the ability of the model to exclude false positive cases, was significantly better for RF (1.0) and LR (0.979) than SVM (0.936) and DT (0.83). The PPV of DT (0.826) and LR (0.972) are consistent with their sensitivity, which indicates that the predictions are well balanced. External validation confirmed sustained efficacy, with SVM and LR maintaining high sensitivity, accuracy, and AUC. LR exhibited particularly stable specificity retention.

**Table 2:** Diagnostic performance across test and external validation sets.

Model	AUC (95% CI)		Sensitivity (95% CI)		Specificity (95% CI)		PPV (95% CI)		NPV (95% CI)		Accuracy	
	Internal-testing set	External-verification	Internal	External	Internal	External	Internal	External	Internal	External	Internal	External
RF	0.857 [0.772–0.941]	0.788 [0.624–0.951]	0.761 [0.621–0861]	0.533 [0.301–0.752]	1.0 [0.924–1]	1.0 [0.806–1]	1.0 [0.901–1]	1.0 [0.676–1]	0.810 [0.901–1]	0696 [0.491–0.844]	0.882	0.774
LR	0.876 [0.798–0.953]	0.858 [0.726–0.990]	0.761 [0.621–0.861]	0.80 [0.548–0.930]	0.979 [0.889–0.996]	0.813 [0.570–0.934]	0.972 [0.858–0.995]	0.80 [0.548–0.930]	0.807 [0.687–0.889]	0.813 [0.570–0.934]	0.871	0.806
SVM	0.894 [0.819–0.969]	0.879 [0.751–1.0]	0.804 [0.668–0.893]	0.933 [0.702–0.988]	0.936 [0.828–0.978]	0.688 [0.444–0.858]	0.925 [0.801–0.974]	0.737 [0.512–0.882]	0.830 [0.708–0.908]	0.917 [0.646–0.985]	0.871	0.806
DT	0.828 [0.750–0.905]	0.644 [0.470–0.818]	0.826 [0.693–0.909]	0.60 [0.357–0.802]	0.830 [0.699–0.911]	0.688 [0.444–0.858]	0.826 [0.693–0.909]	0.643 [0.388–0.837]	0.830 [0.699–0.911]	0.647 [0.413–0.827]	0.828	0.645

**Figure 6:**
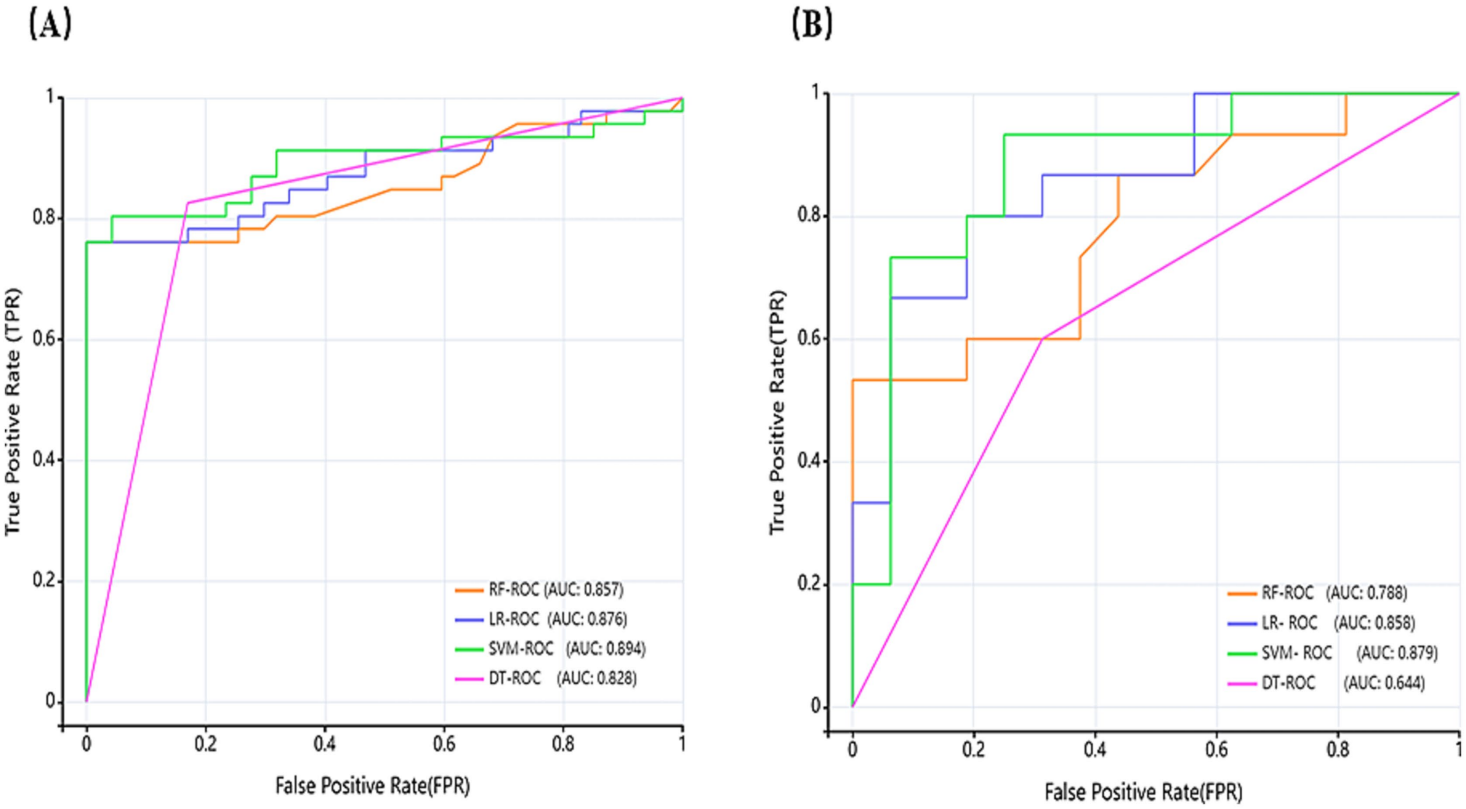
Receiver operating characteristic (ROC) curves of models **(A)** depicts the ROC of the test set, and **(B)** depicts the ROC of the external validation set.

ROC curves were generated to comparatively assess model classification performance (Figure 6). Test set analysis (Figure 5A) revealed SVM achieved optimal overall discrimination (AUC = 0.894), with its curve closest to the upper-left corner, significantly outperforming other models. LR demonstrated secondary performance (AUC = 0.876). RF curve (AUC = 0.857) was in the middle, but had extremely high specificity (1.0), sacrificing some sensitivity. DT showed the lowest AUC (0.828), with its curve nearest the diagonal, yet achieved peak sensitivity (0.826). External validation curves (Figure 5B) exhibited rightward/downward shifts toward the diagonal relative to test set performance. This indicates that the models face some challenges but still ensure notable recognition of HT and hypothyroidism on never-seen external data, and the overall trend remains consistent with the test set, with some generalization ability.

**Figure 7:**
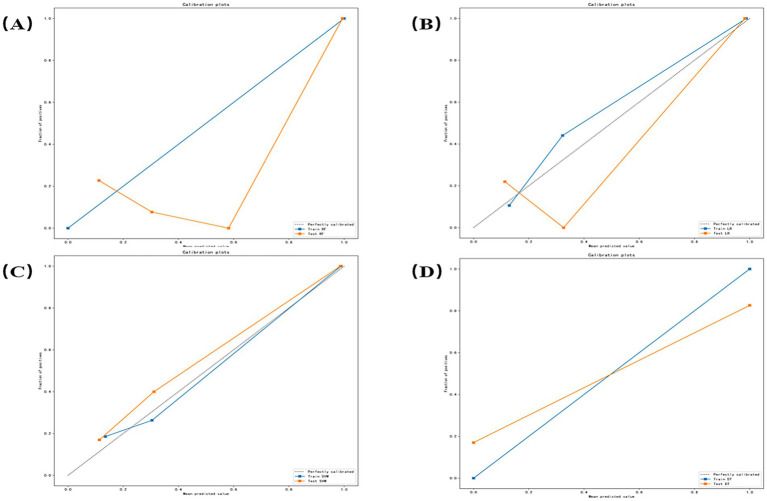
Calibration curves for each model **(A–D)** correspond, respectively, to the random forest (RF), logistic regression (LR), support vector machine (SVM), and decision tree (DT) models.

Calibration curves were generated to evaluate prediction reliability and generalizability for distinguishing euthyroid versus hypothyroid HT subgroups (Figure 7). Figures 7A–D correspond, respectively, to the RF, LR, SVM, and DT models. Orange curves represent test set performance. RF exhibited significant underprediction at probabilities >0.6 and minor over prediction in low-risk zones (<0.3). LR demonstrated optimal alignment, nearly overlapping the diagonal between 0.4 and 0.7, with minor deviations only at extremes (<0.2 or >0.8). SVM showed systematic underprediction across all probability ranges with a stable offset. DT demonstrates a stepwise deviation.

## 4 Discussion

Hashimoto’s thyroiditis (HT) has become the most common autoimmune disease and one of the leading causes of hypothyroidism in developed countries (21). Although histopathology remains the diagnostic gold standard, fine-needle aspiration biopsy (FNAB) demonstrates limited screening utility due to its invasive nature, operator dependence, and poor patient acceptance, particularly for benign conditions or population screening. Current diagnosis integrates clinical manifestations, serological testing, and ultrasound structural changes (22)^,^ yet serological methods remain cost-prohibitive for routine screening, while ultrasound exhibits limited diagnostic accuracy. Consequently, an urgent need exists for non-invasive, cost-effective screening tools with robust diagnostic performance to facilitate early HT/hypothyroidism detection and longitudinal monitoring.

In recent years, artificial intelligence (AI) has made breakthroughs in medical image analysis and intelligent-assisted diagnosis, bringing technological innovation to traditional tongue diagnosis. As a non-invasive, repeatable modality reflecting systemic physiological status, TCM tongue diagnosis offers unique advantages. The introduction of machine learning has shifted tongue diagnosis toward standardization and objectivity, while retaining the traditional advantages as a non-invasive, low-cost, and convenient tool. Algorithmic processing, such as image segmentation, color calibration, and texture extraction, substantially reduces acquisition biases from equipment, illumination, and operator variability (23, 24). Machine learning is broadly divided into two paradigms: deep learning and traditional machine learning. Compared to deep learning, traditional machine learning models, which rely on feature engineering, offer superior interpretability. This characteristic is crucial for integrating tongue image-based AI with traditional Chinese medicine theory, as it enables clinicians to understand the decision logic of the model, thereby facilitating clinical acceptance of AI-assisted diagnostics. Furthermore, traditional machine learning models incur lower computational costs for both training and inference, making them more suitable for deployment within the hardware constraints typically found in primary healthcare settings. Given these advantages. In recent years, the research on tongue images based on machine learning algorithms has increased. Jiang et al. (25) developed SVM, RF, and GBDT (boosted decision tree) models for tongue image quality control. Zhang et al. (26) established an SVM-based diabetes diagnosis model using standardized tongue images. What is more, Li et al. (27) confirmed tongue features significantly enhance diabetes risk prediction accuracy in ML models. Previous studies on machine learning in tongue diagnosis have mainly focused on diagnostic consistency and reducing human subjective bias, while the research on tongue recognition for autoimmune diseases, such as HT, is still in its infancy. This study applies traditional machine learning to explore the value of tongue images in HT diagnosis. Enabling scalable, standardized image databases for AI-driven disease identification and staging models.

We analyzed standardized tongue images from patients with HT and its accompanying hypothyroidism, extracting high-dimensional features via machine learning to quantify disease-state recognition value. Notably, stringent adherence to inclusion and exclusion criteria during the image acquisition phase was essential to establish a comparable foundation for tongue image feature modeling and to minimize confounders such as medication use and comorbidities that may affect tongue appearance. Moreover, subtle factors including dietary habits and oral hygiene may influence microbial flora or induce coating discoloration, thereby altering tongue image presentation. Previous studies have suggested that marker bacteria associated with different tongue coating types can vary across diseases (28). Although efforts were made to control for known confounders, factors such as dietary habits and oral hygiene, which were not fully controlled, may still exert subtle influences on tongue images. Therefore, future studies could further optimize the model by collecting more comprehensive clinical data, including detailed medical interviews and lifestyle habit surveys. Following preprocessing, tongue images acquired under the current inclusion/exclusion criteria retained discriminative features across texture, margin, chromaticity, and morphology. Analysis of top-ranking features indicated that “contrast” (from GLCM), as well as “area” and “perimeter” (from Shape2D descriptors), contributed substantially to classification performance. The results demonstrate that the GLCM value of hypothyroidism patients is significantly higher, indicating an increase in the complexity of tongue texture, which may be related to the thickening and uneven distribution of the tongue; prominent Shape2D features suggested hypothyroidism patients’ tongue hypertrophy and marginal irregularity; the change of the First Order is significant, which corresponds to the change of the tongue color shades, indicating that the tongue color of the hypothyroidism patients is pale or cyanosis; while glszm (gray-scale region size matrix) and gldm (gray-scale dependency matrix) is relatively weak, indicating that their contribution to the classification is limited. These alterations align with hypothyroid pathophysiology (e.g., slowed basal metabolism, water and fluid retention, and slowed circulation), which are prominent in the thermograms, validating the ability of the AI model to perceive the pathological features of the tongue.

t-SNE dimensionality reduction confirmed that the tongue features could effectively differentiate between normal and hypothyroid individuals in high-dimensional space. The euthyroid and hypothyroid groups demonstrated a discernible separation trend in the two-dimensional projection, indicating the model’s strong discriminatory capability. However, some boundary blurring and overlapping clusters were observed. These overlapping samples likely correspond to patients in the subclinical or early stages of the disease, where tongue manifestations are not yet typical. In such cases, single-modality tongue image features may be insufficient to form distinct clusters, representing a limitation in model identification. Potential solutions for future research directions include: firstly, developing a multimodal fusion model by integrating tongue image data with serological indicators (e.g., antibody levels, TSH) and ultrasonographic features. This approach could leverage the complementary information from different data modalities, thereby improving the accuracy of identifying early-stage or subclinical disease. Secondly, conducting prospective cohort studies to periodically collect tongue images from euthyroid HT patients and monitor their thyroid function changes could explore whether alterations in tongue appearance serve as early warning indicators for predicting progression to hypothyroidism.

Comprehensive analysis of the above visualization results demonstrates that tongue image features such as texture uniformity and roughness of tongue moss, size and edge contour of the tongue body, and color depth of the tongue body are important discriminative indicators for distinguishing euthyroid and hypothyroid. Therefore, this is highly consistent with TCM diagnostic principles of “observing the color, examining the shape, and identifying the moss.” Which further validates the feasibility and effectiveness of quantitative tongue features in AI-driven diagnostic modeling.

In terms of model construction, all four ML models demonstrated great discriminative capacity for HT and hypothyroidism classification (AUC > 0.82), and models were stable in differentiating ability (CI lower bounds >0.75). What is more, SVM achieved optimal AUC (0.894), significantly outperforming RF (0.857), LR (0.876), and DT (0.828), indicating superior classification stability. DT showed peak sensitivity (0.826; 95% CI upper: 0.909), making it ideal for identifying true-positive cases and suitable for primary screening scenarios, while SVM followed (0.804), with RF/LR lowest (0.761). Specificity indicates the model’s ability to exclude false positive cases, with RF (1.0) and LR (0.979), significantly exceeding SVM (0.936) and DT (0.830). CI lower bounds of RF (0.924) is still higher than other models with the strongest reliability. RF and LR achieved highest PPV (1.0 and 0.972, respectively), ensuring maximal reliability for positive predictions. The PPV and sensitivity of DT (0.826) and LR (0.972) are consistent with each other, indicating that the predictions are well balanced. In summary, SVM emerged as the optimal balanced model (AUC = 0.894, sensitivity = 0.804, specificity = 0.936), which best reconciled recall and precision requirements. The 95% CI of all metrics did not overlap (e.g., 0.798–0.953 for LR and 0.819–0.969 for SVM), indicating that the differences between models were statistically significant.

In clinical practice, a reliable auxiliary diagnostic model requires both excellent classification accuracy and trustworthy predicted probabilities. The former is measured by the Area Under the Curve (AUC), while the latter can be assessed through calibration. According to the calibration curve (Figure 7), the good calibration of the Logistic Regression (LR) model indicates that its predicted risk probabilities (e.g., 50, 70%) closely align with the actual observed disease prevalence. This is crucial for clinical decision-making, as it enables physicians to perform more accurate risk stratification and patient communication based on the model’s specific probability outputs—for instance, determining the necessity for further serological or ultrasonographic examination. In contrast, although the Support Vector Machine (SVM) model achieved the highest AUC (0.894), its systematic miscalibration could lead to an underestimation of patient risk by clinicians.

The external validation set is an important criterion for assessing the generalization ability of the models. Although this study was conducted within a Chinese population, data were specifically collected from two medical centers in different provinces (Liaoning and Anhui), and an independent external validation set was employed to assess the model’s generalizability. All models retained significant HT/hypothyroidism recognition capacity in external validation, mirroring test set performance trends. SVM maintained superior performance (AUC = 0.879) with peak sensitivity (0.933) and NPV (0.917), demonstrating robust diagnostic capability and strong clinical translation potential. LR achieved balanced performance (AUC = 0.858, sensitivity = 0.800, specificity = 0.813, accuracy = 0.806), showing stable clinical utility through optimal sensitivity-specificity equilibrium. RF preserved perfect specificity and PPV but exhibited significant sensitivity degradation (0.533) and reduced accuracy (0.774), indicating overfitting susceptibility, which reflected a certain risk of overfitting. Risk. DT demonstrated limited generalizability needs to be improved (AUC = 0.644, accuracy = 0.645).

There are still some limitations in this study. Firstly, while our single-center modeling and multi-center external validation preliminarily established the diagnostic utility of tongue imaging for HT/hypothyroidism, broader validation across diverse populations and regions is required to enhance generalizability and clinical implementation. The next step is to conduct a more mature external validation in multi-center, multi-region to further train the generalizability of the model and optimize the benefits of the model in the actual clinical workflow. To enhance the validity of our findings, future investigations will incorporate a prospective design, multi-center collaboration, and a formal *a priori* power calculation to ensure adequate statistical power, thereby improving the robustness and generalizability of the conclusions. Furthermore, although this study focused on classifying patients with HT by developing a binary classifier for thyroid functional status, future work incorporating healthy controls or non-HT hypothyroid patients (e.g., those with hypothyroidism due to iodine deficiency or post-thyroidectomy) to construct a multi-class model would further broaden its clinical applicability.

## 5 Conclusion

Early detection of HT and associated hypothyroidism is critically significant in improving patient prognosis and implementing individualized management. We developed and validated four AI diagnostic models using multidimensional tongue imaging features. The results demonstrate that Tongue features demonstrated significant discriminative capacity for euthyroid and hypothyroid, and machine-learning-assisted tongue image possesses feasibility and validity in screening HT and its concomitant hypothyroidism, which still maintains a notable performance in an independent external validation. These findings establish tongue imaging as a novel noninvasive biomarker for HT diagnosis and risk stratification. In the future, joint modeling with multimodal medical data is expected to improve the diagnostic accuracy and provide a new path for intelligent screening and individualized management of thyroid diseases.

## Data Availability

The original contributions presented in the study are included in the article/supplementary material, further inquiries can be directed to the corresponding authors.
